# Development of Eco-Friendly Concrete Mix Using Recycled Aggregates: Structural Performance and Pore Feature Study Using Image Analysis

**DOI:** 10.3390/ma15082953

**Published:** 2022-04-18

**Authors:** Plaban Deb, Barnali Debnath, Murtaza Hasan, Ali S. Alqarni, Abdulaziz Alaskar, Abdullah H. Alsabhan, Mohammad Amir Khan, Shamshad Alam, Khalid S. Hashim

**Affiliations:** 1 Department of Civil Engineering, Chandigarh University, Mohali 140413, Punjab, India; plaban930@gmail.com; 2 Department of Civil Engineering, North Tripura District Polytechnic, Bagbassa 799253, Tripura, India; brnali540@gmail.com; 3 Department of Civil Engineering, College of Engineering, King Saud University, P.O. Box: 800, Riyadh-11421, Saudi Arabia; abalaskar@ksu.edu.sa (A.A.); aalsabhan@ksu.edu.sa (A.H.A.); salam@ksu.edu.sa (S.A.); 4 Department of Civil Engineering, Galgotia College of Engineering, Knowledge Park I, Greater Noida 201310, Uttar Pradesh, India; amirmdamu@gmail.com; 5 Built Environment and Sustainable Technologies (BEST) Research Institute, Liverpool John Moores University, Liverpool L3 3AF, UK; k.s.hashim@ljmu.ac.uk

**Keywords:** recycled brick aggregate, pervious concrete, image processing, pore feature

## Abstract

The shortage of natural aggregates has compelled the developers to devote their efforts to finding alternative aggregates. On the other hand, demolition waste from old constructions creates huge land acquisition problems and environmental pollution. Both these problems can be solved by recycling waste materials. The current study aims to use recycled brick aggregates (RBA) to develop eco-friendly pervious concrete (PC) and investigate the new concrete’s structural performance and pore structure distributions. Through laboratory testing and image processing techniques, the effects of replacement ratio (0%, 20%, 40%, 60%, 80%, and 100%) and particle size (4.75 mm, 9.5 mm, and 12.5 mm) on both structural performance and pore feature were analyzed. The obtained results showed that the smallest aggregate size (size = 4.75 mm) provides the best strength compared to the large sizes. The image analysis method has shown the average pore sizes of PC mixes made with smaller aggregates (size = 4.75 mm) as 1.8–2 mm, whereas the mixes prepared with an aggregate size of 9.5 mm and 12.5 mm can provide pore sizes of 2.9–3.1 mm and 3.7–4.2 mm, respectively. In summary, the results confirmed that 40–60% of the natural aggregates could be replaced with RBA without influencing both strength and pore features.

## 1. Introduction

The growing need for natural aggregates in the construction sector is causing a scarcity of materials and creating an environmental imbalance. [1,2]. Some countries, therefore, have restricted the excess use of natural aggregates in construction [3]. The north-eastern part of India, especially Tripura, is suffering from the unavailability of natural aggregates, and these are mostly imported from the nearby country Bangladesh [4,5,6]. As the states do not have their own source of natural aggregates and depend on other states/countries, the cost of natural stone is much higher in such areas [7]. As the world is becoming environmentally more conscious, any project nowadays not only considers the economy of the project but also checks the environmental impact that the project would have on the livelihood of the human being. Being a nonrenewable resource, natural aggregates requires an alternative to counteract their overuse and shortages [8]. On the other hand, demolishing old structures produces a considerable amount of construction and demolition waste (CDW) that requires expensive disposal, and thereby large land areas become occupied [9,10]. Billions of tons of CDW annually are generated worldwide, causing severe environmental problems, such as water and soil pollution and infrastructural problems. For example, choking sewer systems in Chennai, India, due to the accumulation of CDW, resulted in a severe flood in 2015 [11]. Therefore, researchers are constantly trying to find innovative ways to recycle or reuse CDW to minimize their severe environmental and infrastructural problems effects, such as using recycled aggregates (RA) and recycled concrete aggregates (RCA) in construction material [7,8,9]. The concept of using RA as a construction material is not new, and there are several studies on the use of RA in concrete mixes or asphalt mixes [10,11,12,13,14,15,16,17]. In addition, researchers have recently tried to use RA in pervious concrete (PC) mixes [18,19].

Pervious concrete (PC) is an emerging construction concept that is a benefit to urban developers as it is the best and most sustainable way to control urban stormwater [20,21,22]. Currently, most developed and many developing countries are looking forward to using the PC in urban areas for its advantages from an ecological and hydrological point of view. Researchers have studied several aspects of PC as a pavement structure for managing heavy runoff in urban areas and ‘Urban Heat Island’ control [23,24,25,26]. PC is mainly porous concrete mixes having a certain amount of voids through which the runoff can be transferred to the lower layers and finally to the groundwater. Around 15–30% of voids are generally present in the PC mixes that can effectively produce an infiltration rate of around 0.1–3.5 cm/s [20,27]. However, the presence of these voids drastically affects the strength of PC mixes [28,29]. Hence, pervious concrete pavements cannot be implemented on any good quality service roads such as expressways, highways, or any other connecting roads. The major application of PCs can be found in parking lots, sidewalks, shoulders, etc., where low-strength pavements are also acceptable. The compressive strength of PC mixes generally ranges from 2 to 30 MPa, whereas the average strength of ‘Pavement Quality Concrete (PQC)’ is around 40 MPa. The foregoing studies on PCs have incorporated several governing factors such as (a) size, shape, and type of aggregate, (b) mix proportions, (c) void content, (d) water–cement ratio, (e) aggregate–binder ratio, (f) compaction type, and many others.

Zaetang et al. [30] have used lightweight aggregates (LWA) for preparing PC mixes and observed that the strength of PC mixes using LWA ranged from 2.47 MPa to 6 MPa. Considering the type of aggregates, the majority of the researchers have used natural stone aggregates, although some researchers have used lightweight aggregates, recycled aggregates, brick aggregates, steel slag aggregates, etc. [19,28,30,31,32,33,34,35]. Debnath and Sarkar [36] carried out a detailed characterization of PCs using over-burnt bricks and found that brick aggregates can be a good option for minimizing the consumption of stones. Steel slag aggregates were also used in the production of PC; the results of these studies indicated the compressive strength of the produced PC is in the range of 10–30 MPa with a permeability range of 0.2–2.8 cm/s [33,37]. Gaedicke et al. [38] found that using RA in PC could keep the compressive strength of the produced PC (PC-RA) in the range of 8–20 MPa, whereas the compressive strength of PC made of natural aggregates (PC-NA) is about 28 MPa. In addition, the permeability of the PC-RA is in the range of 0.5–1.5 cm/s, whereas the maximum permeability of PC-NA is 1.2 cm/s. Other studies on PC-RA have also mentioned that RAs are feasible enough to be used in PC mixes. However, all the previous studies focused on recycled concrete aggregates (RCA) and they have not focused particularly on recycled brick aggregates (RBAs). Thus the performance and suitability of RBAs in pervious concrete mixes are unknown. The present study addresses this research gap and aims at using recycled brick aggregates (RBA) in the production of PC. This study mostly focuses on the mechanical properties of the produced PC and the suitability of RBAs in PC mixes. Moreover, the past studies on PC-RA mainly conducted experimental tests to find out the performances, while the current study uses image analysis along with the experimental tests to investigate the performance of the produced concrete. The novelty of this research work mainly lies in the use of RBA in the PC mixes which will help to mitigate the shortage of NA as well as the nuisances created by CDW. This study primarily provides a simple overview of the structural behavior and pore feature details of PC mixes prepared with recycled brick aggregates.

## 2. Experimental Plan

### 2.1. Aggregates and Other Materials

**Figure 1:**
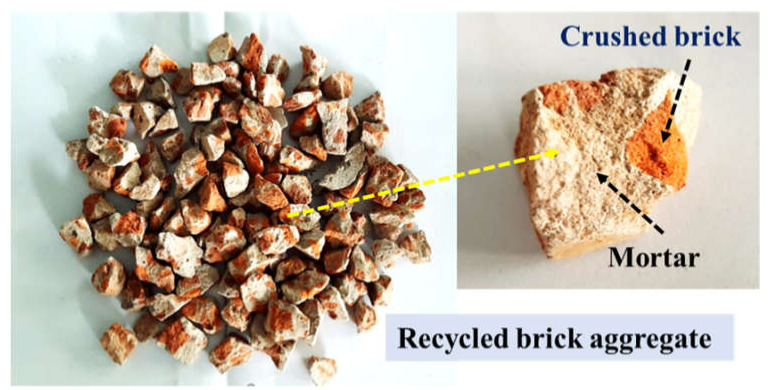
Recycled brick aggregate (RBA).

Demolition wastes were collected from a nearby construction site in India, and then recycled brick aggregates were collected from these, see Figure 1. The latter shows that the aggregates consist of crushed brick and mortar. The collected aggregates were sieved and graded to obtain three different aggregate gradations named RBA-12.5, RBA-9.5, and RBA-4.75, see Table 1. The general properties of RBAs are also evaluated and compared with the properties of natural stone and natural brick aggregates (NBA) (Table 2). The table provides the average values of different test results. The properties were calculated for different size fractions and then the average values are represented here for ease of comparison. Ordinary Portland Cement (OPC)-43 grade cement was used as a binder for preparing PC mixes, and sand was used as fine aggregate. The use of sand in PC mixes is generally prohibited, but some studies mentioned that sand provides better bonding and is essential when non-conventional aggregates are used in PC mixes [22]. Thus, in this study, 10% of sand by volume of coarse aggregate is used in the mix.

#### Mix Design and Preparation of Samples

The addition of RBA in PCs was performed in different sets depending upon the quantity of RBA. Several blending proportions were used, and natural aggregate (NA) was partially/fully replaced by RBA. The blending proportions (NA:RBA) were 100:0, 80:20, 60:40, 40:60, 20:80, and 0:100. The mix design method used in this study was following the research conducted by Debnath and Sarkar [36], as there is a lack of standard specifications for mix design of PC with non-conventional aggregates. The water–cement ratio was chosen as 0.3, and the designed air void was 20%. Table 3 shows the mixed proportions of cement, aggregate, and water along with a commercial superplasticizer (BASF Master Rheobuild 1125, Master Builders Solutions India Private Limited, Navi Mumbai, Maharashtra, India). The samples were prepared in a traditional concrete mixer, and then the prepared mix was transferred into some prefabricated molds depending upon the type of tests. A curing period of 28 days was fixed for all the mixes.

## 3. Analysis Methods

### 3.1. Structural Behavior

**Figure 2:**
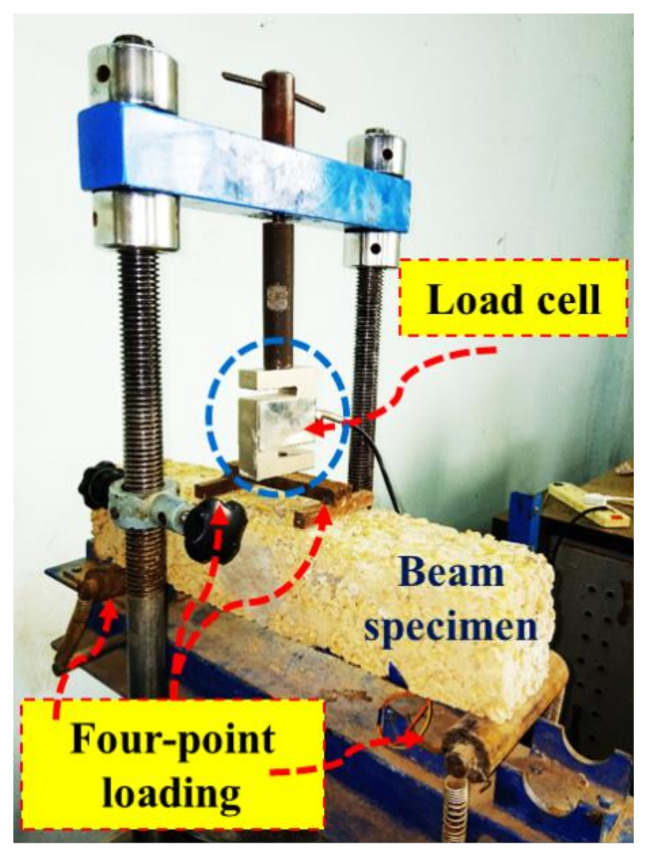
Four-point bending test.

The structural performance of the PC was evaluated by checking its compressive strength and flexural strength behavior. According to IS: 516 [41], the compressive strength was performed using a cube of 150 mm × 150 mm × 150 mm. The cubes were prepared using the standard cube molds followed by IS:516 [41]. The loading rate during testing was kept as 4 tonne/mm. The flexure behavior of PC was calculated using beams of size 100 mm × 100 mm × 500 mm. A four-point loading system was used for this test, as shown in Figure 2, where the load was applied through a loading frame, and the failure load was recorded using a load cell.

#### Pore Feature and Image Analysis

**Figure 3:**
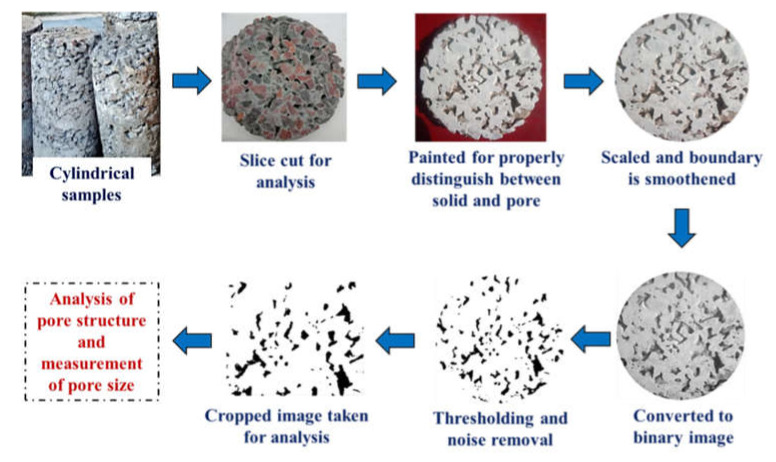
Method of image processing.

The porous nature of PC was accumulated through porosity analysis in both the experimental and image analysis process. The experimental method adopted for porosity analysis was followed by ASTM C1754 [42] and other past studies [28]. Cylinders of 100 mm in diameter and 200 mm in height were chosen for performing the tests, where the basic water-displacement concept was used. In the 2nd phase, the PC mixes were analyzed using the popular ‘Image Processing Technique (IPT)’, where the PC images were processed and thresholded to find the number of voids present in the mixes. Image processing is a widely used method in different fields of engineering. For the past few years, this IPT is also being used for identifying several responses of structures [43]. Freely available software ‘*ImageJ^TM^*’ was used here, and the stereological analysis was conducted [44]. The method adopted for IPT is described through a flow chart in Figure 3. Initially, circular images were scanned from different locations of different PC samples. A similar cylindrical specimen was used for IPT as used for porosity calculation. Then the specimen was cut at five equal intervals to find thin slices, which means each specimen gave five slices. Three replicates were also used for each mix to quantify the variability in the pore distributions, which ultimately produced 15 slices for a particular mix type. The slices were then painted white to identify pores and solid phases properly. Once the solid and pores were identified, the slices were then scanned with a flatbed scanner, and the images’ scaling was performed. After scaling and smoothening of boundaries, these were converted into binary images followed by thresholding and unnecessary noise removal. Finally, a square cross-section was cropped from each image (equal size for each type of mix), which denoted the representative area element (RAE). This RAE signifies the behavior of a whole specimen, and hence this RAE should be selected carefully. As PC mixes are porous and have variability in pore distribution, 15 RAEs were taken from each mix, which can represent the behavior of that particular mix.

## 4. Results and Discussions

### 4.1. Strength Behavior of PC-RBA

#### 4.1.1. Compressive Strength

**Figure 4:**
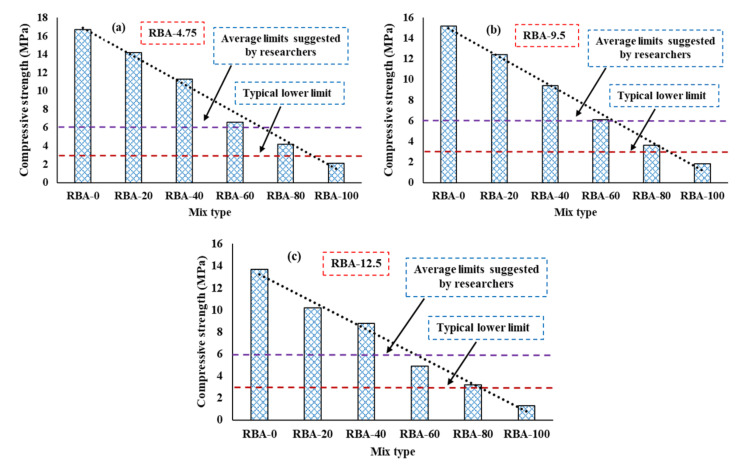
Compressive strength of PC mixes, (**a**) for RBA-4.75, (**b**) RBA-9.5, (**c**) RBA-12.5.

This section gives the detailed test results of compressive strength (fcs) of PC mixes produced with RBAs. The variation of fcs values for different blending proportions of RBA is shown in Figure 4, where it can be observed that the average fcs values are becoming reduced with the increased proportions of RBA. The PC mix with 100% NA has shown an fcs value of 14–16.7 MPa, whereas the fcs value of the PC mix with 100% RBA is only about 1.3–2.1 MPa. The recycled bricks have lesser strength properties such as crushing value, impact value, etc., compared to natural ones (refer to Table 2), and hence the concrete mixes produced with RBA become weaker than PC-NA. The typical lower limit of fcs for PC mixes is recommended as 3 MPa [45] and the majority of researchers have found or suggested an fcs value of at least 6 MPa for PC mixes made with single-sized aggregates [30,46]. However, it is observed in the figure that the PC mix with 100% RBA is not able to provide the lower limit of fcs, and can give an fcs value of about 1.3–2.1 MPa, irrespective of aggregate size. The PC mix with 80% RBA can produce an fcs value of about 3.2 MPa, 3.6 MPa, and 4.2 MPa for RBA-12.5, RBA-9.5, and RBA-4.75, respectively higher than the lower limit, although these do not satisfy the suggested limit (6 MPa). However, the PC mix made with 60% RBA is showing an fcs value of 6.1 MPa and 6.6 MPa for RBA-9.5 and RBA-4.75, which is very slightly higher than the suggested limit, while the mix RBA-60 with an aggregate size of 12.5 mm can reach an fcs value of 4.9 MPa. On the other hand, the PC mixes with 40% or 20% can provide fcs values much higher than the suggested limit for any aggregate size. For the mixes RBA-40 and RBA-20, the fcs values are 1.4–1.8 times and 1.7–2.4 times higher than the suggested limit. Therefore, if 6 MPa is taken as a benchmark of compressive strength for PC mixes, the natural aggregates can be replaced by 60% of RBA, when the aggregate size is 9.5 mm or less. Similarly, for an aggregate size of 12.5 mm, the natural aggregates can be replaced by 40% of RBA.

#### 4.1.2. Flexural Strength

**Figure 5:**
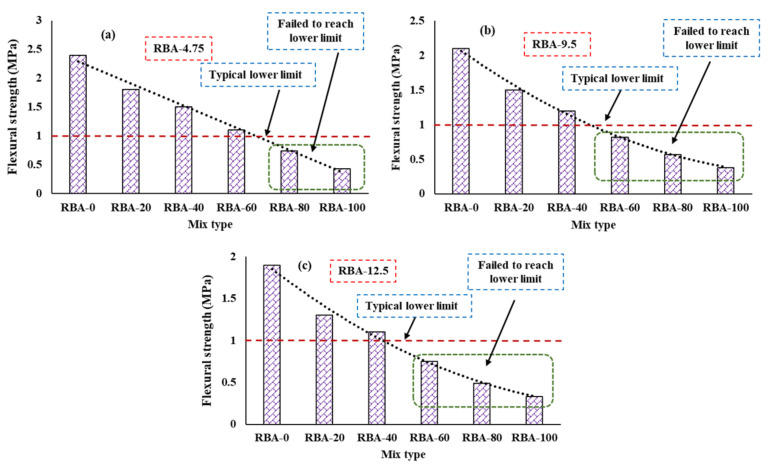
Flexural behavior of PC, (**a**) for RBA-4.75, (**b**) RBA-9.5, (**c**) RBA-12.5.

As pervious concretes are mostly used as paved sections, the flexural response also becomes essential. The flexural strength (fr) variations for different mixes are shown in Figure 5, where it is observed that the mixes produced with a higher percentage of RBA are not suitable to withstand a high flexural load. The variations of fr are somewhat similar to that of fcs due to the poor strength of RBAs. The path of flexural load transfer mostly propagates through the aggregates, and hence the weaker RBAs cannot take more loads. The typical lower limit of fr was suggested as 1 MPa [26,39], and it is found that the PC mixes made with 100% and 80% RBA cannot provide the minimum required value of fr for RBA-4.75. When the 4.75 mm aggregate is used for preparing PC mixes, fr values are obtained as 0.43 MPa, 0.74 MPa, 1.1 MPa, 1.5 MPa, 1.8 MPa, and 2.4 MPa for the mixes RBA-100, RBA-80, RBA-60, RBA-40, RBA-20, and RBA-0, respectively. However, for RBA-9.5 and RBA-12.5, 100% and 80% and 60% RBA usage are not useful for obtaining a limit value of flexural strength. The aggregate size of 9.5 mm fr values are obtained as 0.38 MPa, 0.57 MPa, 0.82 MPa, 1.2 MPa, 1.5 MPa, and 2.1 MPa for the mixes RBA-100, RBA-80, RBA-60, RBA-40, RBA-20, and RBA-0, respectively. Similarly, the aggregate size of 12.5 mm fr values are obtained as 0.33 MPa, 0.49 MPa, 0.75 MPa, 1.1 MPa, 1.3 MPa, and 1.9 MPa for the mixes RBA-100, RBA-80, RBA-60, RBA-40, RBA-20, and RBA-0, respectively. Another important thing that can be noticed here is that the value of fr is higher for smaller aggregates compared to larger aggregates, which is primarily happening due to the presence of smaller pore sizes. The gap between two large aggregates is much higher than the gap between two small aggregates, and hence the crack propagation in the mixes with larger aggregates becomes much easier.

### 4.2. Pore Feature Analysis

**Figure 6:**
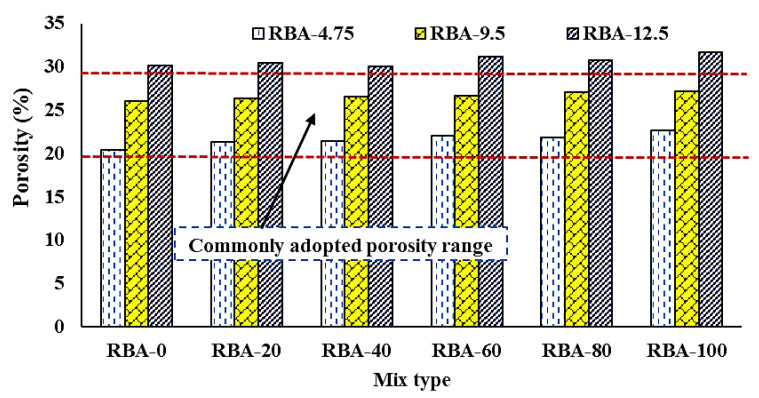
Variation of porosity in PC mixes.

A suitable porous structure and sufficient amount of voids are the key factors of PCs as an adequate water percolation rate can only be achieved due to the presence of these voids. In this study, the porous nature is checked in two ways: (a) porosity measurement and (b) stereological analysis through IPT. The experimental porosity results are shown in Figure 6, where it can be found that the effect of RBAs on the change of porosity values is very little as the porosity value is slightly increasing with the increase in RBA content. It was observed from the literature that the porosity of PC mixes ranges from 15–30%, and here all the mixes show a porosity range of 20% to 30%, which is quite satisfactory. However, the difference in porosity values is much more prominent if the size of aggregates is changed. For an aggregate size of 4.75 mm, the porosity lies in the range of 20–22%, increasing up to 26–27% if a 9.5 mm size aggregate is used. Similarly, the porosity values can be achieved up to 32% if the aggregate size is kept at 12.5 mm. Such varying porosity is common because of the production of different sizes of pores in PC mixes when varying aggregates are used.

**Figure 7:**
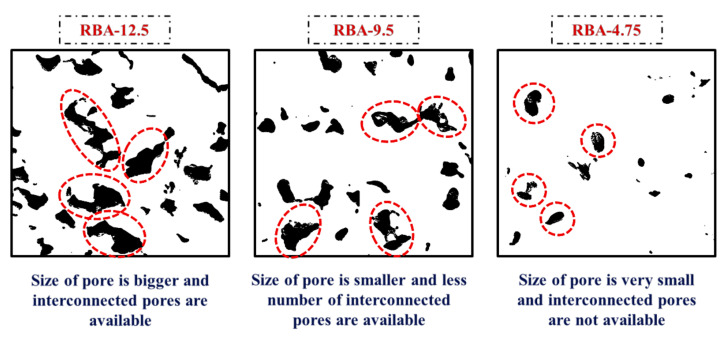
Typical RAEs for mixes with different aggregate sizes.

**Figure 8:**
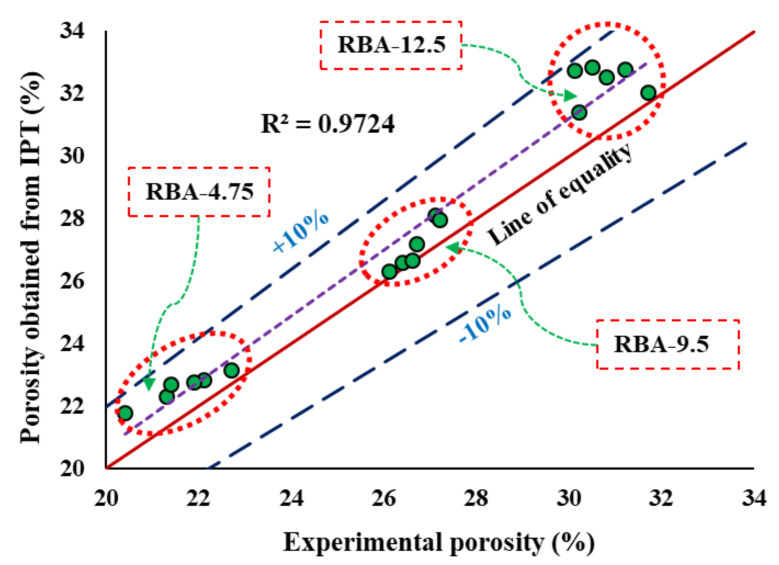
Comparison of experimental porosity with the porosity obtained from IPT.

This can be better understood from the RAEs collected from different mixes, reflecting the pore distribution in a particular PC mix (Figure 7). Image analysis helps to understand the size of the pores and the distribution of pores in a mix. In this study, the size of pores is identified from the stereological method, which signifies that the area fractions present in each RAE will be numerically equal to the actual porosity calculated experimentally. From this stereological image analysis, several parameters are obtained, such as the area fractions, pore histograms, and pore size present in each type of mix. The area fractions identified for each slice of each replicate of all mixes are mentioned in Table 4, along with the standard deviations and the experimental porosity values. The standard deviation values obtained for the slices S1 to S15 for each type of mix lie in the range of 0.76–1.97. This indicates that the replicates of each mix do not show any great variability and the pore distribution along the specimen is almost homogeneous. According to the stereological theory, the average values of *A_f_* need to be the same as *n_exp_*. However, it is found that the values of *A_f_* for each mix are slightly higher than *n_exp_*, indicating a higher void content (Figure 8). However, this variation lies in the range of 0.2% to 7%, and hence it can be assumed that the chosen RAEs can easily replicate the actual specimen.

**Figure 9:**
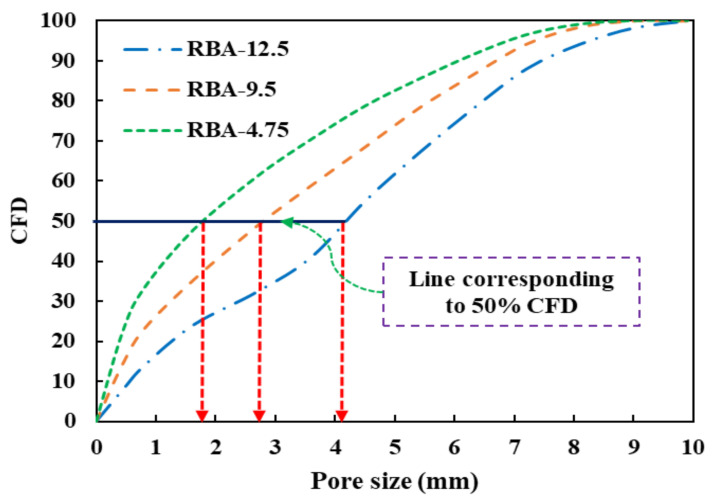
Frequency distribution and pore size calculation.

**Figure 10:**
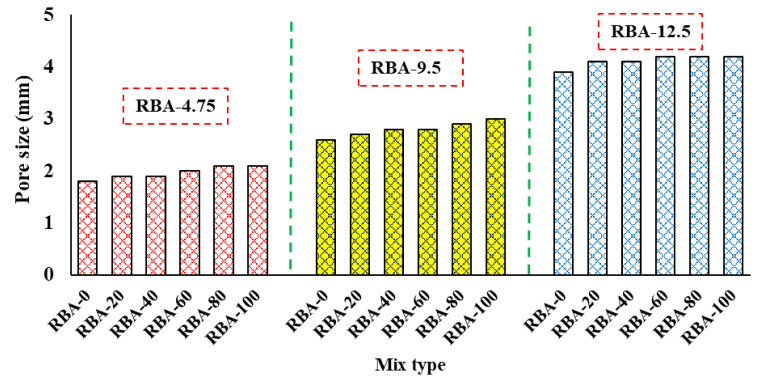
Average pore sizes for different mixes.

Another major outcome obtained from IPT is calculating the size of the pores in each mix. For obtaining the size of pores, two assumptions are taken in this study, (a) the shapes of the pores are circular, and (b) each pore has a distinct property. Several pore sizes are obtained from a single RAE, and then the cumulative frequency distribution (CFD) curves are drawn to find the equivalent pore size. In the present study, the equivalent pore size is taken as the *d_50_* value, i.e., the pore size matching with 50% CFD curve is considered as *d_50_*. The variation of CFD curves for different mixes is shown in Figure 9, and the average pore sizes for all the mixes are mentioned in Figure 10. It can be seen that the size of the pores for the mixes produced with RBA-12.5 is much larger than that of the mixes produced with RBA-4.75, which is mainly attributed to the presence of larger-sized aggregates in the mixes of RBA-12.5. It can also be understood from Figure 7, where the processed images are shown for three different sizes of aggregates. The available pore size for RBA-4.75 is much smaller as compared to RBA-12.5.

## 5. Summary of the Test Results

This study provides a preliminary understanding of the usage of recycled brick aggregates in the preparation of pervious concrete mixes. Several percentages of RBAs replace the natural aggregates, and the structural performance, i.e., the strength of the PC mixes, is checked through experimental tests. Being a bi-functional structure, pervious concrete also possesses internal voids for adequate water percolation. This study checks this porous nature through experiments and image processing techniques. The major outcome of the study deals with the applicability of RBAs in PC, and it is found that the full replacement of natural aggregates may not be possible as the effects of RBAs and NAs in PC mixes are not the same. If a PC mix is produced with 100% NA, the compressive strength can be obtained up to 14–16.7 MPa, which will be reduced to 1.3–2.1 MPa with the usage of 100 RBAs. The overall results show that the natural aggregates can be replaced by up to 40–60% of RBAs for ensuring a satisfactory strength. However, the aggregate size also plays an important role here as the smaller aggregates can provide better strength than large aggregates. Compressive and flexural strength values show that 60% of RBAs can be used in PCs if the aggregate size is chosen as 4.75 mm. However, if the aggregate size is larger than 4.75 mm (9.5 mm or 12.5 mm), natural aggregates can be replaced by 40% of RBAs. The porosity does not have much effect on the quantities of RBA that are being used in the mix; rather the porosity is greatly affected by the size of the RBA used in the mix. It is found that the use of 12.5 mm sized RBAs can provide a better porosity, although the use of 4.75 mm sized RBAs can also provide a satisfactory porosity. However, the image analysis reveals that the size of the pores present in PC mixes made with 12.5 mm sized aggregate is about 80% higher than that of the mixes made with 4.75 mm sized aggregates. Considering both strength and porosity, it can be decided that the size of RBA used in PC should be in the range of 4.75 mm to 9.5 mm. In short, it is clear from the test results that the RBAs can easily replace a certain percentage of NA, which will help to conserve our natural resources and also minimize the adverse environmental effects of demolition wastes.

## 6. Conclusions

The deficiency of natural aggregates for the growing demand for construction is assisting the search for alternate materials. From the perspective of natural resource conservation, other marginal materials have more environmental benefits. Moreover, the generation of substantial demolition waste creates several environmental nuisances that can only be minimized by recycling or reusing the waste materials. Both these severe issues can be curtailed by using recycled aggregates in the construction work, which will resolve the problems associated with waste disposal and reduce the consumption of natural aggregates. This study uses recycled brick aggregate in pervious concrete mixes with the intention of partially or fully replacing the natural aggregates. However, the test results disclose that the use of 100% RBAs in PC mixes may not be suitable from the structural point of view, as the use of 100% RBAs cannot provide adequate strength for the PC mixes. The overall test results and image analysis show that the smaller-sized RBAs (4.75 mm to 9.5 mm) are very useful for providing adequate pore size and sufficient strength and porosity. Although some laboratory tests and image analyses of the PC samples have been conducted in this study, further studies need to be carried out for the overall application of RBA in PC. The performance of PC under fatigue load needs further study because the pavements are often subjected to repeated loading and are more prone to fatigue failure.

## 7. Limitations and Future Scope

Although some laboratory tests and image analyses of the PC samples have been conducted in this study, further studies need to be carried out for the overall application of RBA in PC. This study is primarily focused on a particular design mix of PC for several percentages of NA and RBA. It is assumed that the mix could be resolved by optimizing the mix proportions, and several trials could be conducted to obtain an optimum mix design. This can be considered a limitation of this study and can be studied further. Moreover, the durability study and cost analysis of PC with RBA require future analysis for the practical application of PC with RBA. Although the cost analysis has not been performed in this study, the use of recycled materials will be more economical in some locations due to the high price of stones in those areas. The performance of PC under fatigue load also needs further study because the pavements are often subjected to repeated loading and are more prone to fatigue failure. The life cycle cost analysis, fatigue studies, etc., are the future research scopes of this study, which can also be conducted to understand the applicability of RBAs in PC.

## Figures and Tables

**Table 1:** Aggregate gradation.

Gradation	Size of Aggregate (Percentage Passing)				
	13.2 mm	12.5 mm	9.5 mm	6.3 mm	4.75 mm
RBA-12.5	100	0	0	0	0
RBA-9.5	100	100	0	0	0
RBA-4.75	100	100	100	100	0

**Table 2:** General properties of RBA (standard specifications were followed from [39,40]).

Properties	NA	RBA	NBA (Over-Burnt)	Standard Guidelines
Impact value (%)	18.4	33.4	36.2	IS:2386, Part IV [40]
Abrasion value (%)	25.6	40.8	45.3	IS:2386, Part IV [40]
Crushing value (%)	22.5	36.5	38.7	IS:2386, Part IV [40]
Specific gravity	2.781	1.975	1.912	IS:2386, Part III [39]

**Table 3:** Mix proportion of PC mixes.

Mix Type	NA (%)	RBA (%)	NA (kg/m^3^)	RBA (kg/m^3^)	Binder (kg/m^3^)	Sand (kg/m^3^)	Water (kg/m^3^)	Admixture (kg/m^3^)
RBA-0	100	0	1619.57	0	242.42	161.83	72.73	1.94
RBA-20	80	20	1295.66	230.12	242.42	161.83	72.73	1.94
RBA-40	60	40	971.74	460.24	242.42	161.83	72.73	1.94
RBA-60	40	60	647.83	690.36	242.42	161.83	72.73	1.94
RBA-80	20	80	323.91	920.48	242.42	161.83	72.73	1.94
RBA-100	0	100	0	1150.60	242.42	161.83	72.73	1.94

**Table 4:** Details of area fractions obtained from RAEs.

Mix Type	R-1					R-2					R-3					St Dev	Average	Experimental Porosity (*n_exp_*)
	S1	S2	S3	S4	S5	S6	S7	S8	S9	S10	S11	S12	S13	S14	S15			
RBA-0	21.4	24.2	21.6	22.4	25.4	23.2	18.7	20.8	21.7	24.3	20.5	23.4	21.1	18.3	20.1	1.97	21.81	20.4
RBA-20	22.4	23.6	20.7	19.7	21.4	23.7	26.2	21.5	20.6	25.4	23.4	20.2	19.7	23.6	22.4	1.94	22.30	21.3
RBA-40	21.6	22.4	22.3	24.6	22.7	23.4	21.8	22.6	25.1	23.2	20.6	22.1	21.9	23.6	22.7	1.11	22.71	21.4
RBA-60	23.6	25.1	20.4	22.7	23.4	21.8	26.7	21.4	25.3	24.1	21.3	20.2	21.4	23.6	21.4	1.86	22.83	22.1
RBA-80	21.5	22.3	21.7	24.1	23.5	24.3	22.4	22.9	21.6	23.4	21.4	25.1	22.2	23.6	21.7	1.13	22.78	21.9
RBA-100	22.6	25.4	23.1	22.3	21.6	24.8	22.7	23.3	21.6	22.9	24.7	23.5	24.1	23.5	21.4	1.17	23.17	22.7
RBA-0	28.4	25.3	25.6	24.1	25.3	27.4	24.6	25.8	26.8	26.9	27.8	25.6	28.9	26.8	25.7	1.34	26.33	26.1
RBA-20	26.4	25.5	26.5	27.3	27.7	26.3	26.9	25.8	26.1	25.5	26.2	27.4	26.7	26.2	28.4	0.79	26.59	26.4
RBA-40	27.4	26.8	26.7	25.8	27.4	28.3	25.7	26.8	25.9	26.3	27.4	25.9	27.3	26.4	25.7	0.76	26.65	26.6
RBA-60	26.7	28.6	25.6	28.4	27.6	25.7	28.3	27.4	26.8	25.8	26.3	25.8	29.1	27.4	28.4	1.15	27.19	26.7
RBA-80	27.8	28.9	26.7	29.4	28.4	27.6	28.3	28.7	28.9	26.8	27.7	28.8	29.6	27.4	26.8	0.91	28.12	27.1
RBA-100	29.4	27.6	28.7	28.3	28.4	27.5	29.5	27.4	28.5	26.7	28.7	26.7	25.9	27.5	28.8	1.00	27.97	27.2
RBA-0	31.2	32.5	29.8	30.7	30.6	31.6	31.7	31.5	30.2	30.8	33.4	31.4	32.1	31.6	32.2	0.90	31.42	30.2
RBA-20	32.4	33.6	32.7	33.4	31.5	33.8	31.2	32.4	32.8	33.6	34.6	33.4	32.8	31.8	32.7	0.89	32.85	30.5
RBA-40	33.2	32.6	32.1	32.8	33.2	36.4	33.2	31.5	31.4	32.3	31.9	32.7	32.5	33.4	31.7	1.16	32.73	30.1
RBA-60	33.5	31.6	32.7	32.5	32.4	33.6	33.8	31.5	34.2	31.7	32.6	31.8	32.7	33.8	33.4	0.86	32.79	31.2
RBA-80	32.7	32.8	32.1	30.8	34.5	32.2	32.6	31.6	33.4	32.6	32.8	31.9	33.5	31.9	32.5	0.84	32.53	30.8
RBA-100	34.3	32.1	31.5	30.6	32.1	31.6	30.7	31.8	31.1	32.5	32.2	33.1	30.8	34.6	31.4	1.16	32.03	31.9
